# 2-Amino-4-ferrocenyl­thia­zole

**DOI:** 10.1107/S2056989022007228

**Published:** 2022-07-19

**Authors:** Bertin Anzaldo, Pankaj Sharma, Claudia. P. Villamizar, Rene Gutierrez Perez, Rubén A. Toscano

**Affiliations:** a Instituto de Química, Circuito Exterior Cd. Universitaria, PO Box 04510, Ciudad de México, Mexico; b Lab. Síntesis de Complejos, Fac. Cs. Quím.-BUAP, Ciudad Universitaria, PO Box 156, Puebla, Mexico; Universidad de Los Andes, Venezuela

**Keywords:** crystal structure, ferrocene, thia­zole, amino­thia­zole

## Abstract

The title compound, [Fe(C_5_H_5_)(C_8_H_7_N_2_S)], was synthesized by the direct reaction of acetyl­ferrocene, thio­urea and resublimed iodine. The structure shows one mol­ecule in the asymmetric unit. The amino­thia­zole ring makes an angle of 14.53 (13)° with the ferrocenyl ring to which it is attached. In the crystal, pairs of complex mol­ecules inter­act *via* inter­molecular N—H⋯N hydrogen bonds, forming a cyclic dimer which then inter­acts with other dimers through C—H⋯π inter­actions.

## Abstract 2

The crystal and mol­ecular structure of 2-amino-4-ferrocenyl­thia­zole has been determined. The crystal packing features inter­molecular N—H⋯N and C—H⋯π inter­actions.

## 1. Chemical context

Recently, the synthesis of new hybrid compounds based on a ferrocenyl group linked to a five-membered heterocyclic unit has drawn attention (Sánchez-Rodríguez *et al.*, 2017 ▸; Shao *et al.*, 2006*a* ▸). One important five-membered heterocycle is 2-amino­thia­zole, which is a versatile scaffold extensively used in various branches of chemistry including dyes and in the pharmaceutical industries. 2-Amino­thia­zole derivatives are widely used by medicinal chemists (Das *et al.*, 2016 ▸) and have various applications in medicinal, agriculture and analytical chemistry. They are known to exhibit a wide variety of biological activities such as anti­viral, anti­bacterial, anti­fungal, anti­tubercular, herbicidal and insecticidal (Mishra *et al.*, 2017 ▸; Ji Ram *et al.*, 2019 ▸; Dondoni, 2010 ▸). Thia­zoles are also used as precursors or inter­mediates for the synthesis of a variety of heterocyclic compounds (Zeng *et al.*, 2003 ▸). We report here the crystal and mol­ecular structure of 2-amino-4-ferrocenyl­thia­zole, which has not previously been reported. 
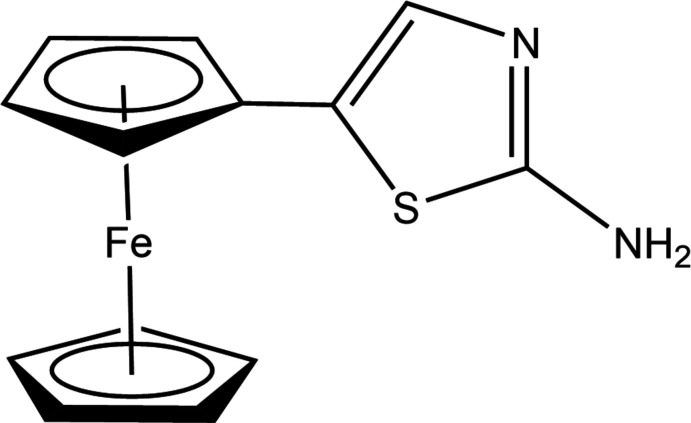


## 2. Structural commentary

**Figure 1:**
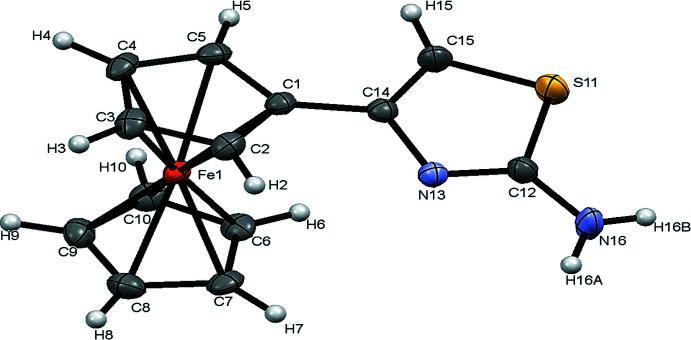
Structure of 2-amino-4-ferrocenyl­thia­zole. Displacement ellipsoids are drawn at the 30% probability level.

The title compound crystallizes in the monoclinic system, space group *P*2_1_/*c*. The asymmetric unit contains one mol­ecular unit as shown in Fig. 1 ▸. The C15—S11—C12 bond angle of 88.6 (2)° reflects the presence of a non-delocalized lone pair of electrons and is similar to that observed in other thia­zoles. The length of the C12=N13 double bond is 1.306 (4) Å. The torsion angles in the amino substituted thia­zole ring are: 1.1 (3)° for N13—C12—S11—C15 and 1.7 (4)° for N13—C14—C15—S11. All bond lengths and angles confirm the *sp* ^2^ hybridization for all C and N atoms.

The ferrocene moiety is in the staggered conformation. The influence of the steric hindrance caused by the organic groups is reflected in the torsion angle C5—C1—C14—C15, 17.0 (5)°, compared with the C2—C1—C14—N13 torsion angle of 13.2 (4)°. The steric effect is also evident in the dihedral angle of 14.77 (17)° subtended by the planes of the heterocycle (C14/C15/S11/C12/N13) and the Cp plane (C1–C5).

## 3. Supra­molecular features

**Figure 2:**
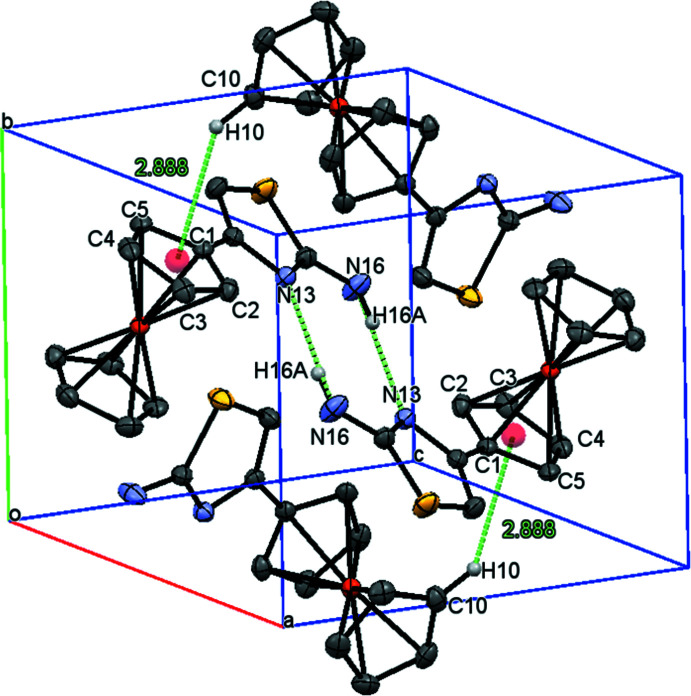
The packing of the title compound. The dotted lines indicate inter­molecular hydrogen bonds. All H atoms not involved in these inter­actions have been omitted for clarity.

The structure is stabilized by inter­molecular hydrogen bonding (N—H⋯N) and C—H⋯π inter­actions. For C10—H10⋯*Cg*(C1–C5) the H-to-ring distance is 2.89 Å, as shown in Table 1 ▸. As a result of inter­molecular N—H⋯N inter­actions, a pseudo six-membered (N16/C12/N13/N16/C12/N13) ring is formed and this hydrogen bond, in addition to the C—H⋯π inter­action, produces a packing into supra­molecular layers parallel to the *bc* plane (Fig. 2 ▸). The structure presents very similar C=N distances and angles in the thia­zole ring, as reported earlier for some similar compounds (Sánchez-Rodríguez *et al.*, 2017 ▸; Shao *et al.*, 2006*b* ▸).

## 4. Database survey

A search of the Cambridge Structural Database (CSD, version 5.43, update of November 2021; Groom *et al.*, 2016 ▸) for 4-ferrocenyl thia­zoles gave eight hits. In six cases (GAVFIT, Yu *et al.*, 2005 ▸; GAVFIT01, Yu *et al.*, 2007 ▸; QAYSAL, Shao *et al.*, 2006*b* ▸; QAYSAL01, Shao *et al.*, 2006*a* ▸; RAPQAB, Shao *et al.*, 2005 ▸; RAPQAB01, Shao *et al.*, 2006*a* ▸), the thia­zole ring is substituted. In two cases there is no substitution in the thia­zole ring (GUPKAG, Xu *et al.*, 2020 ▸ and PAWWEQ, Plazuk *et al.*, 2005 ▸) with PAWWEQ being a diferrocenyl compound. In all eight cases, the bond lengths and angles confirm the *sp^2^* hybridization for all C and N atoms.

## 5. Synthesis and crystallization

The title compound was synthesized according to the reported method (Chopra *et al.*, 2015 ▸). The crude product was purified by column chromatography over silica and suitable crystals were obtained after recrystallization of the solid from a 1:1 hexane-di­chloro­methane mixture by slow evaporation. The compound 2-amino-4-ferrocenyl­thia­zole was further characterized by ^1^H NMR and IR–ATR. FT–IR (ATR, cm^−1^) ν 3099 (ArCH), 2921 (CH_3_), 1658 (C=N); ^1^H NMR (300 MHz, CDCl_3_): 4.62 (2H, *t*, subst. Cp); 4.25 (2H, *t*, subst. Cp); 4.10 (5H, *s*, subst. Cp); 5.00 (2H, –NH_2_), 6.35 (1H, C—H).

## 6. Refinement details

Crystal data, data collection and structure refinement details are summarized in Table 2 ▸. N-bound H atoms were refined isotropically with *U*_iso_(H) = 1.2*U*_eq_(N). C-bound H atoms were positioned geometrically (C—H = 0.93–0.98 Å) and refined with isotropically *U*_iso_(H) = 1.2*U*_eq_(C) using a riding model.

## Supplementary Material

Crystal structure: contains datablock(s) I. DOI: 10.1107/S2056989022007228/dj2046sup1.cif

Structure factors: contains datablock(s) I. DOI: 10.1107/S2056989022007228/dj2046Isup3.hkl

CCDC reference: 1841501

Additional supporting information:  crystallographic information; 3D view; checkCIF report

## Figures and Tables

**Table 1:** Hydrogen-bond geometry (Å, °) *Cg*1 is the centroid of the C1–C5 Cp ring.

*D*—H⋯*A*	*D*—H	H⋯*A*	*D*⋯*A*	*D*—H⋯*A*
N16—H16*A*⋯N13^i^	0.84 (2)	2.14 (2)	2.976 (4)	173 (4)
C10—H10⋯*Cg*1^ii^	0.98	2.89	3.703 (3)	141

Symmetry codes: (i) 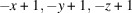 ; (ii) 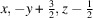 .

**Table 2:** Experimental details

Crystal data	
Chemical formula	[Fe(C_5_H_5_)(C_8_H_7_N_2_S)]
*M*_r_	284.16
Crystal system, space group	Monoclinic, *P*2_1_/*c*
Temperature (K)	298
*a*, *b*, *c* (Å)	14.4024 (4), 7.9621 (2), 10.3584 (3)
β (°)	104.3453 (13)
*V* (Å^3^)	1150.80 (5)
*Z*	4
Radiation type	Mo *K*α
μ (mm^−1^)	1.47
Crystal size (mm)	0.27 × 0.16 × 0.14

Data collection	
Diffractometer	Bruker D8 Venture κ-geometry diffractometer 208039-01
Absorption correction	Multi-scan (*SADABS*; Krause *et al.*, 2015 ▸)
*T*_min_, *T*_max_	0.656, 0.746
No. of measured, independent and observed [*I* > 2σ(*I*)] reflections	17487, 3214, 1805
*R*_int_	0.102
(sin θ/λ)_max_ (Å^−1^)	0.694

Refinement	
*R*[*F* ^2^ > 2σ(*F* ^2^)], *wR*(*F* ^2^), *S*	0.050, 0.091, 1.02
No. of reflections	3214
No. of parameters	160
No. of restraints	1
H-atom treatment	H atoms treated by a mixture of independent and constrained refinement
Δρ_max_, Δρ_min_ (e Å^−3^)	0.44, −0.43

Computer programs: *APEX2* and *SAINT* (Bruker, 2014 ▸), *SHELXT* (Sheldrick, 2015*a* ▸), *SHELXL* (Sheldrick, 2015*b* ▸), *XP* (Siemens, 1998 ▸) and *CIFTAB* (Sheldrick, 2013 ▸).

## supplementary crystallographic information

### Crystal data

**Table:**

[Fe(C_5_H_5_)(C_8_H_7_N_2_S)]	*F*(000) = 584
*M**_r_* = 284.16	*D*_x_ = 1.640 Mg m^−^^3^
Monoclinic, *P*2_1_/*c*	Mo *K*α radiation, λ = 0.71073 Å
*a* = 14.4024 (4) Å	Cell parameters from 5893 reflections
*b* = 7.9621 (2) Å	θ = 2.9–30.0°
*c* = 10.3584 (3) Å	µ = 1.47 mm^−^^1^
β = 104.3453 (13)°	*T* = 298 K
*V* = 1150.80 (5) Å^3^	Prism, orange
*Z* = 4	0.27 × 0.16 × 0.14 mm

### Data collection

**Table:**

Bruker D8 Venture κ-geometry diffractometer 208039-01	3214 independent reflections
Radiation source: micro-focus X-ray source	1805 reflections with *I* > 2σ(*I*)
Helios multilayer mirror monochromator	*R*_int_ = 0.102
Detector resolution: 52.0833 pixels mm^-1^	θ_max_ = 29.6°, θ_min_ = 2.9°
φ and ω–scans	*h* = −19→19
Absorption correction: multi-scan (SADABS; Krause *et al.*, 2015)	*k* = −11→11
*T*_min_ = 0.656, *T*_max_ = 0.746	*l* = −14→14
17487 measured reflections	

### Refinement

**Table:**

Refinement on *F*^2^	Primary atom site location: structure-invariant direct methods
Least-squares matrix: full	Secondary atom site location: difference Fourier map
*R*[*F*^2^ > 2σ(*F*^2^)] = 0.050	Hydrogen site location: mixed
*wR*(*F*^2^) = 0.091	H atoms treated by a mixture of independent and constrained refinement
*S* = 1.02	*w* = 1/[σ^2^(*F*_o_^2^) + (0.024*P*)^2^ + 0.7338*P*] where *P* = (*F*_o_^2^ + 2*F*_c_^2^)/3
3214 reflections	(Δ/σ)_max_ < 0.001
160 parameters	Δρ_max_ = 0.44 e Å^−^^3^
1 restraint	Δρ_min_ = −0.43 e Å^−^^3^

### Special details

**Table:**

Geometry. All esds (except the esd in the dihedral angle between two l.s. planes) are estimated using the full covariance matrix. The cell esds are taken into account individually in the estimation of esds in distances, angles and torsion angles; correlations between esds in cell parameters are only used when they are defined by crystal symmetry. An approximate (isotropic) treatment of cell esds is used for estimating esds involving l.s. planes.

### Fractional atomic coordinates and isotropic or equivalent isotropic displacement parameters (Å^2^)

**Table:**

	*x*	*y*	*z*	*U*_iso_*/*U*_eq_	
Fe1	0.17036 (3)	0.50999 (5)	0.21675 (4)	0.02688 (13)	
C1	0.2361 (2)	0.7055 (3)	0.3316 (3)	0.0293 (7)	
C2	0.2072 (2)	0.5816 (4)	0.4119 (3)	0.0347 (7)	
H2	0.249851	0.519784	0.484765	0.042*	
C3	0.1066 (2)	0.5605 (4)	0.3683 (3)	0.0378 (8)	
H3	0.067801	0.482058	0.405807	0.045*	
C5	0.1513 (2)	0.7608 (4)	0.2371 (3)	0.0354 (8)	
H5	0.148594	0.845906	0.167931	0.043*	
C4	0.0721 (2)	0.6712 (4)	0.2603 (3)	0.0390 (8)	
H4	0.005144	0.683301	0.210031	0.047*	
C6	0.2513 (2)	0.4562 (4)	0.0875 (3)	0.0390 (8)	
H6	0.301224	0.528302	0.067788	0.047*	
C7	0.2651 (2)	0.3335 (4)	0.1884 (3)	0.0401 (8)	
H7	0.326028	0.304522	0.250824	0.048*	
C8	0.1747 (2)	0.2590 (4)	0.1828 (3)	0.0436 (9)	
H8	0.162060	0.169787	0.241401	0.052*	
C9	0.1063 (2)	0.3352 (4)	0.0782 (3)	0.0417 (8)	
H9	0.037655	0.309044	0.051734	0.050*	
C10	0.1538 (2)	0.4568 (4)	0.0194 (3)	0.0388 (8)	
H10	0.123912	0.530116	−0.055375	0.047*	
S11	0.48146 (6)	0.92268 (11)	0.31997 (10)	0.0468 (3)	
C12	0.4914 (2)	0.7406 (4)	0.4136 (3)	0.0340 (7)	
N13	0.41026 (17)	0.6692 (3)	0.4162 (3)	0.0313 (6)	
C14	0.3340 (2)	0.7625 (4)	0.3407 (3)	0.0301 (7)	
C15	0.3588 (2)	0.9019 (4)	0.2849 (3)	0.0407 (8)	
H15	0.315506	0.977392	0.234051	0.049*	
N16	0.5779 (2)	0.6798 (4)	0.4780 (4)	0.0506 (9)	
H16A	0.580 (2)	0.585 (3)	0.514 (3)	0.061*	
H16B	0.627 (2)	0.732 (4)	0.469 (4)	0.061*	

### Atomic displacement parameters (Å^2^)

**Table:**

	*U*^11^	*U*^22^	*U*^33^	*U*^12^	*U*^13^	*U*^23^
Fe1	0.0253 (2)	0.0294 (2)	0.0264 (2)	0.00356 (18)	0.00735 (17)	−0.0012 (2)
C1	0.0303 (17)	0.0283 (16)	0.0294 (18)	0.0051 (12)	0.0075 (14)	−0.0029 (13)
C2	0.0349 (18)	0.0426 (18)	0.0263 (18)	0.0062 (14)	0.0071 (14)	−0.0018 (15)
C3	0.0341 (18)	0.050 (2)	0.035 (2)	0.0000 (15)	0.0180 (15)	−0.0075 (16)
C5	0.0387 (19)	0.0287 (16)	0.036 (2)	0.0100 (14)	0.0042 (16)	−0.0040 (14)
C4	0.0283 (18)	0.048 (2)	0.039 (2)	0.0094 (15)	0.0060 (15)	−0.0144 (17)
C6	0.042 (2)	0.040 (2)	0.042 (2)	0.0015 (14)	0.0252 (17)	−0.0056 (15)
C7	0.0376 (19)	0.0400 (19)	0.044 (2)	0.0147 (15)	0.0119 (16)	−0.0086 (16)
C8	0.056 (2)	0.0291 (17)	0.050 (2)	0.0014 (16)	0.0215 (19)	0.0012 (16)
C9	0.0375 (19)	0.0440 (19)	0.042 (2)	−0.0034 (16)	0.0062 (17)	−0.0154 (17)
C10	0.045 (2)	0.044 (2)	0.0284 (18)	0.0079 (15)	0.0102 (16)	−0.0009 (15)
S11	0.0426 (5)	0.0386 (5)	0.0569 (6)	−0.0050 (4)	0.0081 (4)	0.0175 (4)
C12	0.0351 (18)	0.0308 (16)	0.035 (2)	−0.0025 (14)	0.0062 (15)	0.0041 (14)
N13	0.0284 (14)	0.0299 (13)	0.0348 (16)	0.0007 (11)	0.0064 (12)	0.0043 (12)
C14	0.0329 (17)	0.0277 (16)	0.0289 (18)	0.0020 (13)	0.0060 (14)	−0.0040 (13)
C15	0.0385 (19)	0.0346 (18)	0.045 (2)	0.0035 (14)	0.0029 (16)	0.0112 (16)
N16	0.0283 (16)	0.0447 (18)	0.074 (2)	−0.0057 (13)	0.0045 (16)	0.0265 (17)

### Geometric parameters (Å, º)

**Table:**

Fe1—C6	2.027 (3)	C6—C10	1.408 (4)
Fe1—C7	2.030 (3)	C6—C7	1.408 (4)
Fe1—C8	2.033 (3)	C6—H6	0.9800
Fe1—C5	2.034 (3)	C7—C8	1.419 (4)
Fe1—C2	2.040 (3)	C7—H7	0.9800
Fe1—C4	2.043 (3)	C8—C9	1.408 (4)
Fe1—C1	2.043 (3)	C8—H8	0.9800
Fe1—C10	2.043 (3)	C9—C10	1.409 (4)
Fe1—C3	2.045 (3)	C9—H9	0.9800
Fe1—C9	2.047 (3)	C10—H10	0.9800
C1—C2	1.417 (4)	S11—C15	1.721 (3)
C1—C5	1.432 (4)	S11—C12	1.730 (3)
C1—C14	1.462 (4)	C12—N13	1.306 (4)
C2—C3	1.417 (4)	C12—N16	1.349 (4)
C2—H2	0.9800	N13—C14	1.394 (3)
C3—C4	1.414 (4)	C14—C15	1.340 (4)
C3—H3	0.9800	C15—H15	0.9300
C5—C4	1.416 (4)	N16—H16A	0.84 (2)
C5—H5	0.9800	N16—H16B	0.84 (2)
C4—H4	0.9800		
			
C6—Fe1—C7	40.61 (12)	C4—C3—H3	126.0
C6—Fe1—C8	68.33 (13)	C2—C3—H3	126.0
C7—Fe1—C8	40.88 (12)	Fe1—C3—H3	126.0
C6—Fe1—C5	112.99 (13)	C4—C5—C1	108.4 (3)
C7—Fe1—C5	143.86 (14)	C4—C5—Fe1	70.02 (17)
C8—Fe1—C5	173.68 (13)	C1—C5—Fe1	69.79 (16)
C6—Fe1—C2	131.49 (13)	C4—C5—H5	125.8
C7—Fe1—C2	108.54 (13)	C1—C5—H5	125.8
C8—Fe1—C2	115.77 (13)	Fe1—C5—H5	125.8
C5—Fe1—C2	68.37 (13)	C3—C4—C5	107.9 (3)
C6—Fe1—C4	145.32 (14)	C3—C4—Fe1	69.85 (17)
C7—Fe1—C4	173.96 (14)	C5—C4—Fe1	69.34 (17)
C8—Fe1—C4	135.13 (14)	C3—C4—H4	126.0
C5—Fe1—C4	40.64 (12)	C5—C4—H4	126.0
C2—Fe1—C4	68.26 (12)	Fe1—C4—H4	126.0
C6—Fe1—C1	106.63 (13)	C10—C6—C7	108.3 (3)
C7—Fe1—C1	112.38 (13)	C10—C6—Fe1	70.40 (18)
C8—Fe1—C1	145.08 (13)	C7—C6—Fe1	69.82 (18)
C5—Fe1—C1	41.12 (11)	C10—C6—H6	125.9
C2—Fe1—C1	40.62 (12)	C7—C6—H6	125.9
C4—Fe1—C1	68.84 (12)	Fe1—C6—H6	125.9
C6—Fe1—C10	40.46 (12)	C6—C7—C8	107.5 (3)
C7—Fe1—C10	68.14 (13)	C6—C7—Fe1	69.56 (17)
C8—Fe1—C10	67.91 (13)	C8—C7—Fe1	69.66 (18)
C5—Fe1—C10	108.79 (13)	C6—C7—H7	126.2
C2—Fe1—C10	170.67 (13)	C8—C7—H7	126.2
C4—Fe1—C10	115.84 (13)	Fe1—C7—H7	126.2
C1—Fe1—C10	131.53 (13)	C9—C8—C7	108.1 (3)
C6—Fe1—C3	171.70 (13)	C9—C8—Fe1	70.36 (18)
C7—Fe1—C3	133.85 (14)	C7—C8—Fe1	69.46 (18)
C8—Fe1—C3	111.39 (13)	C9—C8—H8	125.9
C5—Fe1—C3	68.26 (13)	C7—C8—H8	125.9
C2—Fe1—C3	40.58 (12)	Fe1—C8—H8	125.9
C4—Fe1—C3	40.48 (13)	C8—C9—C10	107.9 (3)
C1—Fe1—C3	68.58 (13)	C8—C9—Fe1	69.26 (18)
C10—Fe1—C3	147.70 (13)	C10—C9—Fe1	69.70 (18)
C6—Fe1—C9	68.10 (13)	C8—C9—H9	126.1
C7—Fe1—C9	68.31 (13)	C10—C9—H9	126.1
C8—Fe1—C9	40.38 (12)	Fe1—C9—H9	126.1
C5—Fe1—C9	133.72 (13)	C6—C10—C9	108.2 (3)
C2—Fe1—C9	147.76 (14)	C6—C10—Fe1	69.14 (18)
C4—Fe1—C9	111.42 (13)	C9—C10—Fe1	70.01 (18)
C1—Fe1—C9	171.57 (13)	C6—C10—H10	125.9
C10—Fe1—C9	40.29 (13)	C9—C10—H10	125.9
C3—Fe1—C9	117.46 (14)	Fe1—C10—H10	125.9
C2—C1—C5	106.9 (3)	C15—S11—C12	88.62 (15)
C2—C1—C14	126.6 (3)	N13—C12—N16	123.7 (3)
C5—C1—C14	126.4 (3)	N13—C12—S11	115.2 (2)
C2—C1—Fe1	69.56 (17)	N16—C12—S11	121.1 (2)
C5—C1—Fe1	69.09 (16)	C12—N13—C14	110.0 (3)
C14—C1—Fe1	125.0 (2)	C15—C14—N13	115.2 (3)
C3—C2—C1	108.7 (3)	C15—C14—C1	125.8 (3)
C3—C2—Fe1	69.93 (17)	N13—C14—C1	119.0 (3)
C1—C2—Fe1	69.82 (17)	C14—C15—S11	111.0 (2)
C3—C2—H2	125.6	C14—C15—H15	124.5
C1—C2—H2	125.6	S11—C15—H15	124.5
Fe1—C2—H2	125.6	C12—N16—H16A	118 (2)
C4—C3—C2	108.0 (3)	C12—N16—H16B	118 (2)
C4—C3—Fe1	69.67 (18)	H16A—N16—H16B	123 (3)
C2—C3—Fe1	69.49 (17)		
			
C5—C1—C2—C3	0.0 (3)	C7—C8—C9—C10	−0.2 (4)
C14—C1—C2—C3	−178.4 (3)	Fe1—C8—C9—C10	59.2 (2)
Fe1—C1—C2—C3	−59.3 (2)	C7—C8—C9—Fe1	−59.4 (2)
C5—C1—C2—Fe1	59.2 (2)	C7—C6—C10—C9	0.5 (3)
C14—C1—C2—Fe1	−119.1 (3)	Fe1—C6—C10—C9	−59.3 (2)
C1—C2—C3—C4	0.0 (3)	C7—C6—C10—Fe1	59.8 (2)
Fe1—C2—C3—C4	−59.2 (2)	C8—C9—C10—C6	−0.2 (4)
C1—C2—C3—Fe1	59.2 (2)	Fe1—C9—C10—C6	58.8 (2)
C2—C1—C5—C4	0.0 (3)	C8—C9—C10—Fe1	−58.9 (2)
C14—C1—C5—C4	178.4 (3)	C15—S11—C12—N13	1.1 (3)
Fe1—C1—C5—C4	59.6 (2)	C15—S11—C12—N16	−179.0 (3)
C2—C1—C5—Fe1	−59.5 (2)	N16—C12—N13—C14	179.7 (3)
C14—C1—C5—Fe1	118.8 (3)	S11—C12—N13—C14	−0.4 (3)
C2—C3—C4—C5	0.0 (3)	C12—N13—C14—C15	−0.9 (4)
Fe1—C3—C4—C5	−59.1 (2)	C12—N13—C14—C1	−179.3 (3)
C2—C3—C4—Fe1	59.1 (2)	C2—C1—C14—C15	−165.0 (3)
C1—C5—C4—C3	0.0 (3)	C5—C1—C14—C15	17.0 (5)
Fe1—C5—C4—C3	59.4 (2)	Fe1—C1—C14—C15	105.6 (3)
C1—C5—C4—Fe1	−59.4 (2)	C2—C1—C14—N13	13.2 (4)
C10—C6—C7—C8	−0.6 (3)	C5—C1—C14—N13	−164.9 (3)
Fe1—C6—C7—C8	59.6 (2)	Fe1—C1—C14—N13	−76.2 (3)
C10—C6—C7—Fe1	−60.1 (2)	N13—C14—C15—S11	1.7 (4)
C6—C7—C8—C9	0.5 (4)	C1—C14—C15—S11	180.0 (2)
Fe1—C7—C8—C9	60.0 (2)	C12—S11—C15—C14	−1.6 (3)
C6—C7—C8—Fe1	−59.5 (2)		

### Hydrogen-bond geometry (Å, º)

*Cg*1 is the centroid of the C1–C5 Cp ring.

**Table:**

*D*—H···*A*	*D*—H	H···*A*	*D*···*A*	*D*—H···*A*
N16—H16*A*···N13^i^	0.84 (2)	2.14 (2)	2.976 (4)	173 (4)
C10—H10···*Cg*1^ii^	0.98	2.89	3.703 (3)	141

Symmetry codes: (i) −*x*+1, −*y*+1, −*z*+1; (ii) *x*, −*y*+3/2, *z*−1/2.
